# Retraction notice for: “Long non-coding RNA HOTAIR promotes UVB-induced apoptosis and inflammatory injury by up-regulation of PKR in keratinocytes” [Braz J Med Biol Res (2018) 51(8): e6896]

**DOI:** 10.1590/1414-431X2020e6896retraction

**Published:** 2021-06-14

Guo Liu^1^, Wenhao Zhang^1^

^1^Department of Burns and Plastic Surgery, Jining No.1 People’s Hospital, Jining, Shandong, China

**Retraction for:** Braz J Med Biol Res | doi: http://10.1590/1414-431X20186896 | PMID: 29898032 | PMCID: PMC6002131

The authors would like to retract the article “Long non-coding RNA HOTAIR promotes UVB-induced apoptosis and inflammatory injury by up-regulation of PKR in keratinocytes” that was published in volume 51 no. 8 (2018) (Epub June 11, 2018) of the Brazilian Journal of Medical and Biological Research.

After the publication of this study, the corresponding author requested its retraction due to “the identification of unspecified data inconsistency that could lead to mistaken conclusions.” The Editors agreed with and endorsed that decision.

The Brazilian Journal of Medical and Biological Research had received authorization from all authors before the publication of the paper. We regret the unprofessional behavior of the authors involved.

Correspondence: Wenhao Zhang: <joethomas5725@gmail.com>

